# Nicotinamide Attenuates Complement and Coagulation Pathways and Resultant Renal Fibrosis

**DOI:** 10.1096/fj.202502028R

**Published:** 2025-12-01

**Authors:** Saori Kin, Yuji Oe, Taku Obara, Emiko Sato, Nobuyuki Takahashi, Mariko Miyazaki, Tetsuhiro Tanaka

**Affiliations:** ^1^ Department of Nephrology, Graduate School of Medicine Tohoku University Sendai Japan; ^2^ Division of Preventive Medicine and Epidemiology Tohoku University Tohoku Medical Megabank Organization Sendai Japan; ^3^ Division of Clinical Pharmacology and Therapeutics, Graduate School of Pharmaceutical Sciences Tohoku University Sendai Japan

**Keywords:** inflammasome, NAD^+^, neutrophil extracellular trap, thrombosis

## Abstract

The coagulation and complement pathways interact with each other, promoting inflammation and increasing thrombotic risk. However, their roles in chronic kidney disease (CKD) remain unclear. In our CKD cohort, elevated plasma fibrinogen and shortened prothrombin time were associated with higher serum complement C3 and C4 levels. Moreover, gene expression analysis of CKD renal tissues in public databases showed a positive correlation between fibrinogen β chain and C3 expression levels. Finally, tissue factor (factor III), fibrinogen, and C3 deposition and the genes related to coagulation and complement cascades were upregulated in fibrotic kidneys of mice treated with adenine or folic acid. To explore the modulators of these systems, the therapeutic effects of nicotinamide (NAM), a NAD^+^ precursor with anti‐inflammatory and antithrombotic properties, were investigated. KEGG pathway analysis with RNA sequencing identified a significant inhibition of coagulation and complement cascades by NAM administration in adenine‐induced nephropathy. Moreover, NAM suppressed innate immune responses associated with the prothrombotic state, including inflammasome and neutrophil extracellular trap formation. Collectively, these results suggest that excessive coagulation and complement activities are involved in the pathogenesis of CKD and are modulated by NAM.

## Graphical abstract

The coagulation and complement cascades were elevated in the pathogenesis of chronic kidney disease (CKD) and may contribute to both kidney injury and cardiovascular risk. These excessive signals and the associated innate immune responses were suppressed by the NAD^+^ precursor nicotinamide (NAM). This study identified a novel mechanism of action of NAM that may facilitate its clinical application in the treatment of CKD.

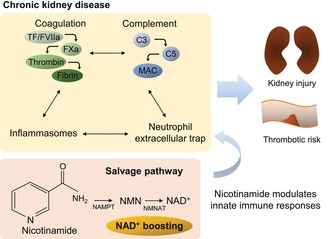

## 1 Introduction

The coagulation and complement systems form part of innate immunity and cross‐talk with each other [1, 2]. The C5 chemotactic fragment or membrane attack complex reportedly increases tissue factor and induces the extrinsic coagulation system, resulting in thrombin formation. Conversely, thrombin can activate the complement pathway by cleaving C3 and C5 [1, 3, 4, 5]. Such excessive complement and coagulation cascades can induce tissue injury via inflammation and thrombotic risks, which are implicated in various diseases such as cardiovascular diseases (CVDs), systemic inflammatory response syndrome, autoimmune diseases, paroxysmal nocturnal hemoglobinuria, and atypical hemolytic uremic syndrome [1, 6]. Recently, complement activation, inflammation, and thrombogenicity have received attention because they contribute to multiple organ failure and the severity of COVID‐19 infection [7, 8].

Hypercoagulability and elevated thrombotic events have been reported in patients with chronic kidney disease (CKD) [9, 10, 11]. Moreover, coagulation proteases induce inflammation and fibrosis via protease‐activated receptors, which contribute to CKD progression [12, 13, 14]. Furthermore, complement factors exacerbate CKD, as local expression of C3 and C5 has been reported to be upregulated in models of kidney fibrosis and to exacerbate fibrosis in the kidneys [15, 16]. Thus, targeting the complement and coagulation cascades can prevent the development of CVDs and progression to end‐stage renal disease and can improve the quality of life of patients with CKD.

We and others have demonstrated the therapeutic effects of nicotinamide (NAM), a precursor of nicotinamide adenine dinucleotide (NAD^+^), in kidney injury models, including acute kidney injury and CKD. NAM is converted to NAD^+^ through a salvage pathway [17] and exerts renoprotective effects by boosting NAD^+^ and reducing inflammation and oxidative stress, activating sirtuin, and maintaining mitochondrial health in kidney injury [18, 19, 20, 21]. More importantly, NAM ameliorates thrombotic microangiopathic lesions in preeclampsia [22, 23], in which the complement and coagulation cascades are activated [24, 25]. Therefore, the mechanism of action of NAM in kidney injury needs to be further elucidated.

This study investigated the link between the prothrombotic state and complement activation in CKD pathogenesis using a CKD cohort, public datasets, and mouse models, with a particular emphasis on the modulatory effects of NAM on these pathways.

## 2 Methods

### 2.1 CKD Cohort in Our Institute

This study was conducted in accordance with the principles of the Declaration of Helsinki and was approved by the Tohoku University Hospital Institutional Review Board (approval no. 2023‐1‐302). Written informed consent was obtained from all participants. The CKD cohort from Tohoku University Hospital included patients who underwent renal biopsy. A total of 179 patients were enrolled from October 2018 to June 2020. The exclusion criteria were as follows: patients who (i) were younger than 20 years; (ii) had rapidly progressive glomerulonephritis, acute tubulointerstitial nephritis, or primary nephrotic syndrome, including minimal change nephrotic syndrome and membranous nephropathy; (iii) met the diagnostic criteria for nephrotic syndrome; (iv) had malignancy, aortic dissection, aortic aneurysm, amyloidosis, or systemic inflammatory diseases (collagen disease, inflammatory bowel disease, sarcoidosis, and IgG4‐related disease); and (v) had missing coagulation and complement related data. Ultimately, 74 patients were included in the analysis.

### 2.2 Human CKD Transcriptomics Datasets

Nephroseq v4 (https://www.nephroseq.org/) and the Ju CKD tubulointerstitium dataset were used to study the expression of coagulation‐ and complement‐related genes and their correlation [26]. To make the background disease in the database and our CKD cohort as identical as possible, we extracted and analyzed data derived from patients “arterial hypertension”, “diabetic nephropathy”, “IgA nephropathy”, “thin basement membrane disease” and “healthy living donor”.

### 2.3 Animal Study

Animal studies were conducted according to the guidelines of Tohoku University and ARRIVE. The experimental protocol was approved by the Institutional Animal Care and Use Committee of Tohoku University (2021MED‐040‐03). C57BL/6J mice aged 8 weeks were obtained from CLEA Japan (Tokyo, Japan). Two mouse models of renal fibrosis were used in this study. One model received a 0.2% adenine‐containing diet (MF diet, Oriental Yeast, Tokyo, Japan) for 2 weeks, after which samples were collected. NAM in drinking water (0.6% in tap water; FUJIFILM Wako Pure Chemical, Osaka, Japan) sufficient to increase renal NAD^+^ levels was also administered at the same time, as previously described [20]. Approximately 1400–1500 mg/kg of NAM was administered to adenine‐treated mice. The second model was folic acid‐induced nephropathy, in which a single intraperitoneal injection of 250 mg/kg folic acid dissolved in sodium bicarbonate solution was administered. The control group received vehicle. Samples were collected 2 weeks later. In addition, to compare the therapeutic effects of NAM and PMX53, a C5aR inhibitor, mice were fed a 0.2% adenine‐containing diet for 1 week to induce renal injury. NAM (0.6% in drinking water), PMX53 (1 mg/kg, intraperitoneal injection; Merk Millipore, Tokyo, Japan), or their combination was administered starting one day before adenine initiation.

### 2.4 Renal Function Assay

Blood urea nitrogen (BUN) and plasma creatinine were measured using a chromogenic assay (Arbor Assays, Ann Arbor, MI, USA) and DRI‐CHEM (FUJIFILM, Tokyo, Japan), respectively. Measurements were performed according to the manufacturer's instructions.

### 2.5 Real‐Time Quantitative PCR

The kidney homogenates were prepared using a bead homogenizer (TOMY, Tokyo, Japan). Total RNA was isolated from whole kidney homogenates using the RNeasy Plus Mini Kit (Qiagen, Germantown, MD, USA) or TRI Reagent (Molecular Research Center, Cincinnati, OH, USA). cDNA was prepared using the iScript Advanced cDNA Synthesis Kit (Bio‐Rad, Hercules, CA, USA) according to the manufacturer's protocol. RT‐PCR was performed using a KAPA SYBR Fast qPCR kit (KAPA Biosystems, Wilmington, MA, USA). The reaction protocols were as follows: initial denaturation at 98°C for 3 min, the reaction was 40 cycles, and each cycle comprised denaturation at 95°C for 5 s and annealing and extension at 60°C for 10 s. *Hprt* was used as the reference gene. The sequence of primers was shown in Table S1.

### 2.6 RNA Sequencing (RNA‐Seq) Analysis

Total RNA was isolated from whole kidney homogenates using an RNeasy Plus Mini Kit (Qiagen, Hilden, Germany). Samples were sequenced on NovaSeq 6000 and this process was performed by Rhelixa Inc. (Tokyo, Japan). Transcript abundance was estimated using salmon against the human transcriptome GRCh39. Gene abundance was then calculated from transcript abundance using the tximport package. Differential expression analysis was performed using the edgeR package after the filterByExpr function was applied to remove low‐expression genes. Genes with |log fold change| > 1 and FDR < 0.05 were considered differentially expressed. These analyses were performed using the OlvTools online web tool (https://olvtools.com/). Gene set enrichment analysis was performed using the ShinyGO 0.81 web tool with an FDR cutoff of < 0.05, displaying the top 20 Kyoto Encyclopedia of Genes and Genomes (KEGG) pathways selected by FDR and ranked by fold enrichment (http://bioinformatics.sdstate.edu/go/) [27]. Heatmaps were generated using the Heatmapper web tool (http://www.heatmapper.ca/) [28]. The raw sequencing data in FASTQ format have been deposited in the DNA Data Bank of Japan (DDBJ) under the accession number PRJDB37909.

### 2.7 Immunohistochemistry

The renal tissues were fixed in 4% paraformaldehyde and embedded in paraffin. They were then cut into 3‐μm‐thick slices for immunohistochemistry. Goat anti‐mouse tissue factor (1:200, #AF3178, R&D Systems, Minneapolis, MN), goat anti‐mouse C3d antibody (1:2000, #AF2655, R&D Systems, Minneapolis, MN, USA), rabbit anti‐human fibrin/fibrinogen antibody (1:4000, #A0080, Dako, Glostrup, Denmark), rabbit anti‐human CD68 (1:2000, #ab125212, Abcam, Cambridge, UK), anti‐rabbit citrullinated histone H3 (CitH3, 1:2000, #97272, Cell Signaling Technology, Danvers, MA), and anti‐human myeloperoxidase (MPO) polyclonal antibody (1:5000, #22225‐1‐AP, Proteintech, Rosemont, IL) were used. Heat‐induced antigen retrieval was performed using sodium citrate buffer for the detection of tissue factor and CitH3, and Dako Target Retrieval Solution (Dako, Glostrup, Denmark) for the detection of MPO. The sections were treated to retrieve antigen using proteinase K (Dako, Glostrup, Denmark) for C3d, CD68, and fibrin/fibrinogen, followed by overnight incubation at 4°C with those primary antibodies. N‐Histofine simple stain kits (Nichirei Biosciences, Tokyo, Japan) were used for secondary antibodies. Sections not incubated with primary antibodies were used as negative controls. To compare the localization of CitH3 and MPO expression, we employed the mirror section method, in which serial sections from the same tissue block are mounted in opposite orientations and stained with different antibodies, allowing for a direct and accurate comparison of the expression sites.

### 2.8 Histological Evaluation

To evaluate histological tubulointerstitial injury, sections were stained with Elastica‐Masson and hematoxylin–eosin. The tubular injury score was semi‐quantified as follows: 0, none; 1, < 10%; 2, 10%–25%; 3, 26%–50%; 4, 51%–75%; and 5, 76%–100%. We measured the ratio of the fibrotic area to the renal cortex area using ImageJ software version 1.54 (NIH, Bethesda, MD; https://imagej.nih.gov/ij). Similarly, the areas positive for tissue factor, fibrinogen, and C3 were measured. For these evaluations, three consecutive fields of the renal cortex were examined on each slide at 100× magnification. The infiltration of CD68, MPO, and CitH3 positive cell counts was scored as follows: 0, none; 1, 1–5 cells; 2, 6–10 cells; 3, ≤ 11 cells/high power field. Approximately 10 non‐overlapping fields per sample were examined, and the individual scores were averaged. All histological evaluations were performed in a blinded manner.

### 2.9 Statistical Analysis

Statistical analyses were performed using JMP Pro 15 (SAS Institute, Cary, NC, USA). Differences were considered statistically significant at *p* < 0.05. The Shapiro–Wilk test was performed to test for normality. In human studies, the results are expressed as mean ± standard deviation (SD) and median with interquartile range for normally and non‐normally distributed variables, respectively. Spearman's rank correlation coefficient was used to assess the linear relationships. Multiple regression analysis was performed to test the effects of complement factors on blood coagulation markers. C3 and C4, age, body mass index (BMI), estimated glomerular filtration rate (eGFR), urinary protein/creatinine ratio, and serum albumin levels were included as explanatory variables to predict plasma fibrinogen and prothrombin time international normalized ratio (PT‐INR). In animal studies, Student's *t*‐test was used for normally distributed data, and log transformation was applied when needed. If normality was not achieved, the Wilcoxon rank‐sum test was performed. Similarly, for comparisons among multiple groups, one‐way ANOVA followed by Tukey–Kramer test was applied to normally distributed data, whereas Kruskal–Wallis followed by Steel–Dwass test was used for non‐normally distributed data, as appropriate. The results are shown as mean ± standard error of the mean (SEM) in animal studies.

## 3 Results

### 3.1 Correlation of Complement and Coagulation‐Related Molecules in Our CKD Cohort

A total of 74 patients were included in the CKD cohort; among these patients, 32, 33, 7, and 2 had stage G1–2, G3, G4, and G5 CKD, respectively. The median age of the patients was 53 (IQR 41–67) years, and the mean eGFR was 59.4 ± 26.8 mL/min/1.73 m^2^. With respect to the coagulation‐related markers, the plasma fibrinogen level, PT‐INR, and activated partial thromboplastin time were 311.3 ± 68.0 mg/dL, 0.98 (median, IQR 0.95–1.01), and 30.5 (median, IQR 28.4–32.6) seconds. Complement C3 and C4 levels were 109.9 ± 20.1 and 27.2 ± 6.9 mg/dL, respectively. The other baseline characteristics are shown in Table S2. Among coagulation markers, fibrinogen levels were negatively correlated with eGFR (*ρ* = −0.306, *p* = 0.009) and PT‐INR values were negatively correlated with high proteinuria (*ρ* = −0.253, *p* = 0.030). Complement C3 and C4 were positively correlated with BMI (*ρ* = 0.427, *p* < 0.001 and *ρ* = 0.297, *p* < 0.001, respectively). More importantly, a statistically significant positive correlation was found between fibrinogen levels and serum C3 and C4 levels (*ρ* = 0.430, *p* < 0.001 and *ρ* = 0.559, *p* < 0.001, respectively). Furthermore, PT‐INR values were negatively correlated with serum C3 and C4 levels (*ρ* = −0.269, *p* = 0.020, and *ρ* = −0.344, *p* = 0.003, respectively) (Table 1). Multiple regression analysis revealed that fibrinogen values correlated significantly with C3 and C4 values after adjusting for age, BMI, eGFR, urinary protein (≥ 0.5 g/gCre), and serum albumin. Similarly, PT‐INR values were significantly correlated with C3 and C4 values after adjusting for other factors (Table 2).

**TABLE 1:** Association between coagulation, complement, and renal function in our CKD cohort.

	Fibrinogen (mg/dL)		PT‐INR		APTT (s)		C3 (mg/dL)		C4 (mg/dL)	
	*ρ*	*p*	*ρ*	*p*	*ρ*	*p*	*ρ*	*p*	*ρ*	*p*
Age	0.226	0.057	−0.169	0.151	0.053	0.652	0.071	0.547	0.018	0.881
BMI (kg/m^2^)	**0.282**	**0.017**	−0.044	0.708	**−0.258**	**0.026**	**0.427**	**< 0.001**	**0.297**	**< 0.001**
U‐protein (g/gCre)	0.226	0.057	**−0.253**	**0.030**	−0.156	0.186	0.167	0.148	0.224	0.055
eGFR (mL/min/1.73 m^2^)	**−0.306**	**0.009**	−0.102	0.387	−0.067	0.572	0.211	0.071	−0.081	0.493
Serum Alb (g/dL)	**−0.246**	**0.038**	0.165	0.160	−0.002	0.985	0.116	0.325	−0.207	0.076
C3 (mg/dL)	**0.430**	**< 0.001**	**−0.269**	**0.020**	−0.092	0.436				
C4 (mg/dL)	**0.559**	**< 0.001**	**−0.344**	**0.003**	−0.221	0.058				

Abbreviations: Alb, albumin; APTT, activated partial thromboplastin time; BMI, body mass index; CKD, chronic kidney disease; eGFR, estimated glomerular filtration rate; Cre, creatinine; PT‐INR, prothrombin time‐international normalized ratio; U‐protein, urinary protein. Bold text indicates statistically significant correlations.

**TABLE 2:** Multiple linear regression analysis of complement‐related molecules for predicting coagulation markers.

	To predict fibrinogen values		To predict log PT‐INR values	
	*β* coefficient	*p*	*β*	*p*
*Model 1*				
C3 (mg/dL)	**0.447**	**< 0.001**	**−0.302**	**0.025**
Log Age	−0.009	0.934	−0.178	0.141
BMI (kg/m^2^)	−0.004	0.974	0.165	0.196
eGFR (mL/min/1.73 m^2^)	**−0.378**	**0.001**	−0.145	0.258
U‐protein (≥ 0.5 g/gCre)	0.038	0.687	**−0.333**	**0.003**
Serum Alb (g/dL)	**−0.333**	**< 0.001**	0.150	0.169
*Model 2*				
C4 (mg/dL)	**0.407**	**< 0.001**	**−0.340**	**0.004**
Log Age	0.098	0.338	**−0.251**	**0.030**
BMI (kg/m^2^)	0.086	0.401	0.122	0.277
eGFR (mL/min/1.73 m^2^)	**−0.209**	**0.046**	**−0.260**	**0.027**
U‐protein (≥ 0.5 g/gCre)	−0.001	0.994	**−0.297**	**0.007**
Serum Alb (g/dL)	**−0.232**	**0.022**	0.081	0.455

*Note:* Model 1: *R* ^2^ = 0.440, adjusted *R* ^2^ = 0.388; Model 2: *R* ^2^ = 0.439, adjusted *R* ^2^ = 0.387 for fibrinogen values. Model 1: *R* ^2^ = 0.257, adjusted *R* ^2^ = 0.190; Model 2: *R* ^2^ = 0.293, adjusted *R* ^2^ = 0.230 for log PT‐INR values. Factors with non‐normal distribution were log‐transformed prior to analysis. Bold text indicates statistically significant correlations.

Abbreviations: Alb, albumin; BMI, body mass index; CKD, chronic kidney disease; eGFR, estimated glomerular filtration rate; Cre, creatinine; PT‐INR, prothrombin time‐international normalized ratio; U‐protein, urinary protein.

### 3.2 Correlation of Coagulation, Complement, and Pro‐Fibrotic Related Genes in Nephroseq

**FIGURE 1:**
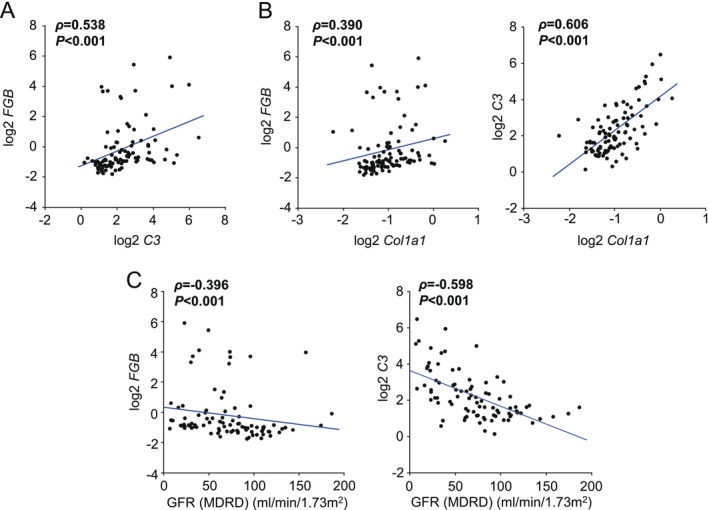
Correlation between renal fibrinogen, C3, and pro‐fibrotic gene expression in the Nephroseq database. (A, B) Correlation among fibrinogen β chain (*FGB*), complement C3 (*C3*), and collagen 1a1 (*Col1a1*) mRNA. (C) Correlation between glomerular filtration rate (GFR) and the expression levels of *FGB* or *C3*. Data were obtained from the Ju CKD tubulointerstitium dataset.

To gain further insights, we used the Nephroseq database to determine the correlation between the expression of coagulation‐ and complement‐related genes in CKD kidney tissues. In the Ju CKD tubulointerstitium dataset, renal expression of *FGB* (fibrinogen β chain) and *C3* (complement C3) in tubules correlated positively with each other (*ρ* = 0.538, *p* < 0.001) (Figure 1A). In addition, *FGB* and *C3* were correlated positively with the expression of profibrotic genes such as *Col1a1* (collagen type I alpha 1 chain) in the kidneys (*ρ* = 0.390, *p* < 0.001 and *ρ* = 0.606, *p* < 0.001, respectively) (Figure 1B). Inversely, *FGB* and *C3* were negatively correlated with GFR (*ρ* = −0.396, *p* < 0.001 and *ρ* = −0.598, *p* < 0.001, respectively) (Figure 1C).

### 3.3 Characteristics of Complement and Coagulation Cascades in Two Models of Renal Fibrosis

**FIGURE 2:**
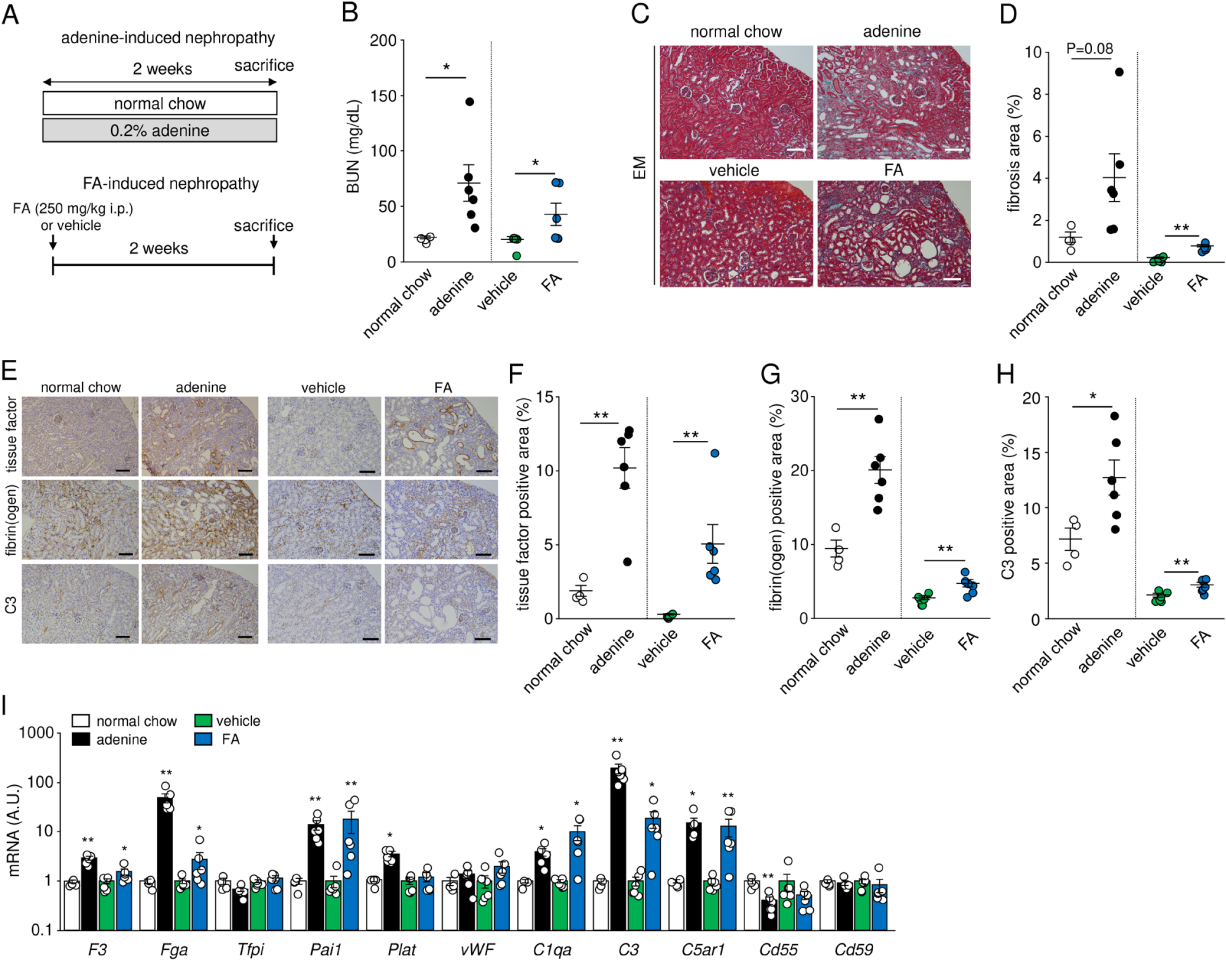
Coagulation and complement cascades were upregulated in two models of renal fibrosis. (A) Experimental protocols of adenine‐ and folic acid (FA)‐induced nephropathy. (B) Blood urea nitrogen (BUN) levels. (C) Photomicrographs of elastica‐Masson (EM) stain in the kidneys. (D) Comparison of renal fibrosis area. (E) Immunohistochemistry of tissue factor, fibrinogen, and C3 in the kidneys. (F–H) Comparison of tissue factor, fibrinogen, and C3 positive area. (I) Gene expression related to coagulation and complement cascades. A.U., arbitrary units. Scale bar = 100 μm. Data are presented as mean ± SEM. *n* = 4–6. *p < 0.05, **p < 0.01.

We next examined whether the changes in complement and coagulation‐related markers in human CKD were also observed in murine renal fibrosis models of adenine and folic acid‐induced nephropathy (Figure 2A). The administration of adenine or folic acid to mice increased BUN and upregulated renal fibrosis (Figure 2B–D). Immunohistochemistry for tissue factor was positive in the vascular smooth muscle and injured tubules, and fibrinogen was prominent in the interstitial areas. Complement C3 level was elevated in the injured tubules and interstitial areas (Figure 2E). When the positive areas were quantified, their expression levels were significantly elevated in adenine‐and folic acid‐induced nephropathy (Figure 2F–H). We further characterized the expression of genes related to the complement and coagulation cascades in the kidneys. In addition to elevated levels of prothrombotic factors such as tissue factor (*F3*), fibrinogen α chain (*Fga*) and *Pai1* gene expression, complement activators *C3*, *C1qa*, and *C5ar1* were upregulated in adenine‐ and folic acid‐induced nephropathy. Interestingly, the gene expression of the complement regulatory protein CD55 (*Cd55*) was significantly downregulated in adenine‐induced nephropathy (Figure 2I).

### 3.4 RNA‐Seq Analysis of Adenine‐Induced Nephropathy Treated With NAM

**FIGURE 3:**
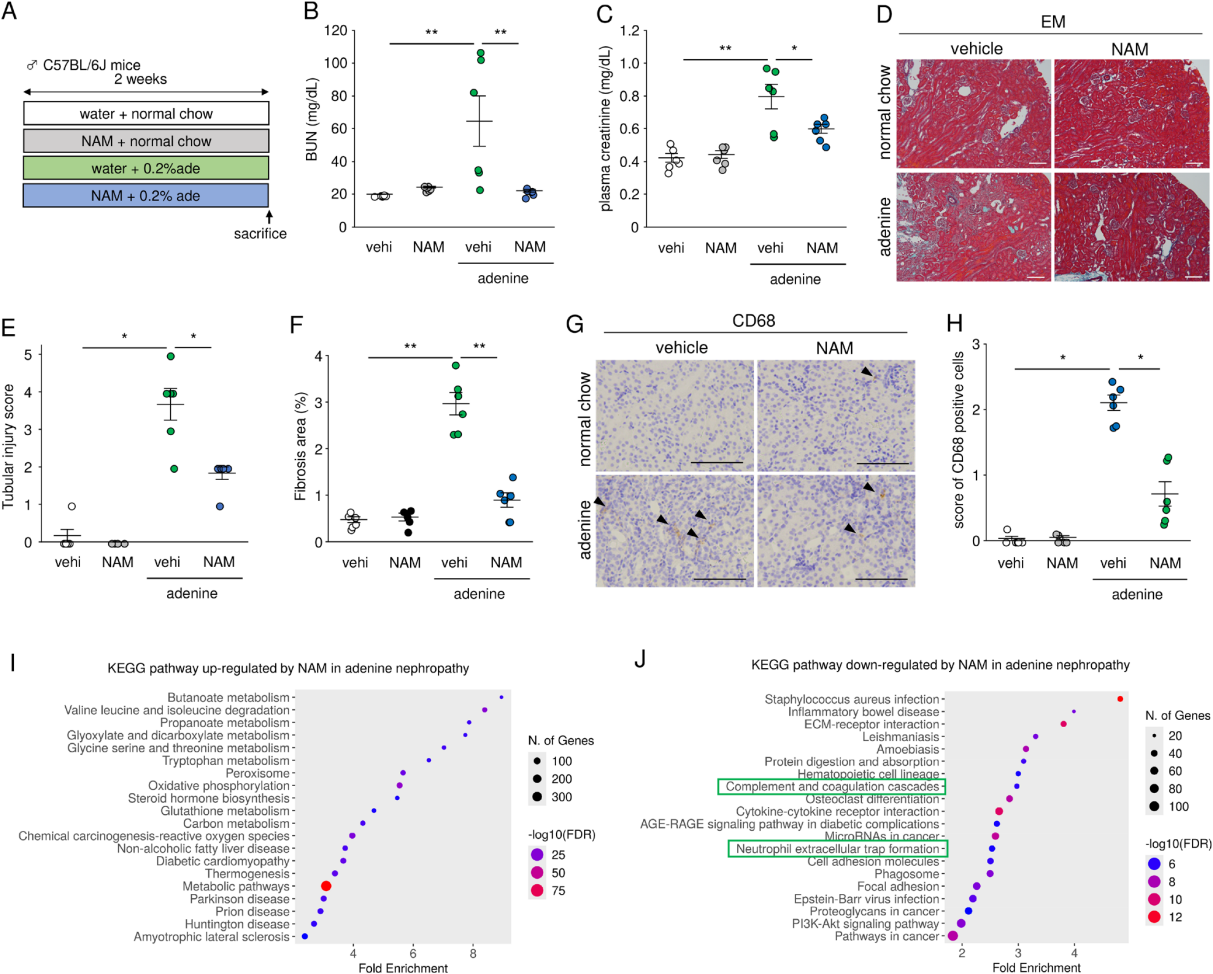
KEGG pathway analysis of alterations by NAM administration in adenine‐induced nephropathy. (A) An experimental protocol. (B, C) Blood urea nitrogen (BUN) and plasma creatinine levels. (D–F) Photomicrographs of elastica‐Masson (EM) stain, along with comparisons of tubular injury scores and renal fibrosis areas. (G, H) CD68 immunohistochemistry in the kidneys and infiltration score of CD68‐positive cells. (I, J) KEGG enrichment analysis of differentially expressed genes in the kidneys. The top 20 pathways are shown. Scale bar = 100 μm. NAM, nicotinamide; vehi, vehicle. Data are presented as mean ± SEM. *n* = 5–6. *p < 0.05, **p < 0.01.

To examine the modulatory effects of NAM on complement‐ and coagulation‐related markers, mice with adenine‐induced nephropathy were treated with NAM (0.6% in drinking water) for 2 weeks (Figure 3A). NAM suppressed elevated BUN and plasma creatinine levels in adenine‐induced nephropathy (Figure 3B,C). Furthermore, NAM improved histological injury, including the tubular injury score and fibrotic area, in adenine‐treated mice (Figure 3D–F). Infiltration of CD68‐positive macrophages was also reduced by NAM (Figure 3G,H). Next, we performed kidney RNA‐seq analysis and identified differentially expressed genes (DEGs) between the control vs. adenine and adenine + NAM versus adenine groups (Figure S1A). Among the overlapping DEGs between these comparisons, we focused on a group of genes that were downregulated by adenine and upregulated by NAM (cluster 1) or upregulated by adenine and downregulated by NAM (cluster 2) (Figure S1B). The results of the KEGG analysis for clusters 1 and 2 are shown in Figure 3I,J. In cluster 1, metabolism‐related pathways, including “butanoate metabolism,” “valine, leucine and isoleucine degradation,” and “propanoate metabolism,” were the top‐ranked pathways. In cluster 2, pathways related to cytoskeletons, chemokines, and fibrosis, such as “ECM‐receptor interaction” and “cytokine‐cytokine receptor interaction,” and pathways related to the innate immune system such as “complement and coagulation cascades” and “neutrophil extracellular trap (NET) formation” were suppressed by NAM (Figure 3I,J). In addition, the NF‐κB pathway, a representative inflammatory signaling pathway, was also significantly enriched and was attenuated by NAM treatment (FDR < 0.0001, fold enrichment = 2.4).

### 3.5 Suppression of the Complement and Coagulation Cascades by NAM in Adenine‐Induced Nephropathy

**FIGURE 4:**
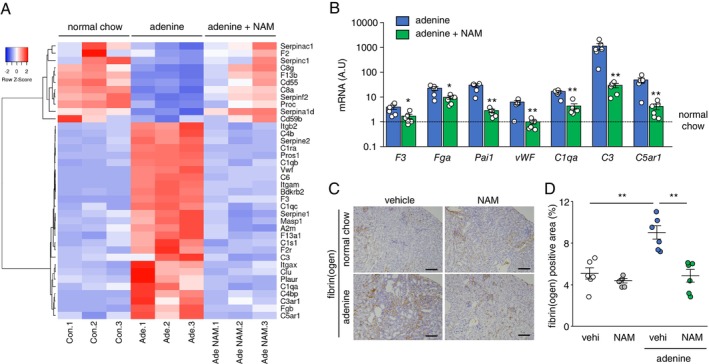
NAM modulated the complement and coagulation cascades in adenine‐induced nephropathy. (A) A heatmap of differentially expressed genes expression related to complement and coagulation cascades. (B) Expression levels of *F3*, *Fga*, *Pai1*, *vWF*, *C1qa*, *C3*, and *C5ar1* mRNA in the kidneys. (C) Photomicrographs of immunohistochemistry against fibrin(ogen). Scale bar = 100 μm. (D) Comparison of fibrin positive area in the kidneys. A.U., arbitrary units; NAM, nicotinamide; vehi, vehicle. Data are presented as mean ± SEM. *n* = 5–6. *p < 0.05, **p < 0.01.

A heat map of the DEGs related to the complement and coagulation cascades is shown in Figure 4A. In addition, the expression changes detected by RNA‐seq were verified by RT‐PCR, and the expression levels of several coagulation‐ and complement‐related genes such as *F3*, *Fga*, *Pai1*, *vWF*, *C3*, *C1qa*, and *C5ar1* were significantly reduced by NAM (Figure 4B). Finally, immunostaining for fibrinogen showed a significant decrease in the positive area in kidneys treated with NAM (Figure 4C,D).

### 3.6 Effect of NAM on Inflammasomes and NET‐Related Markers in Adenine‐Induced Nephropathy

**FIGURE 5:**
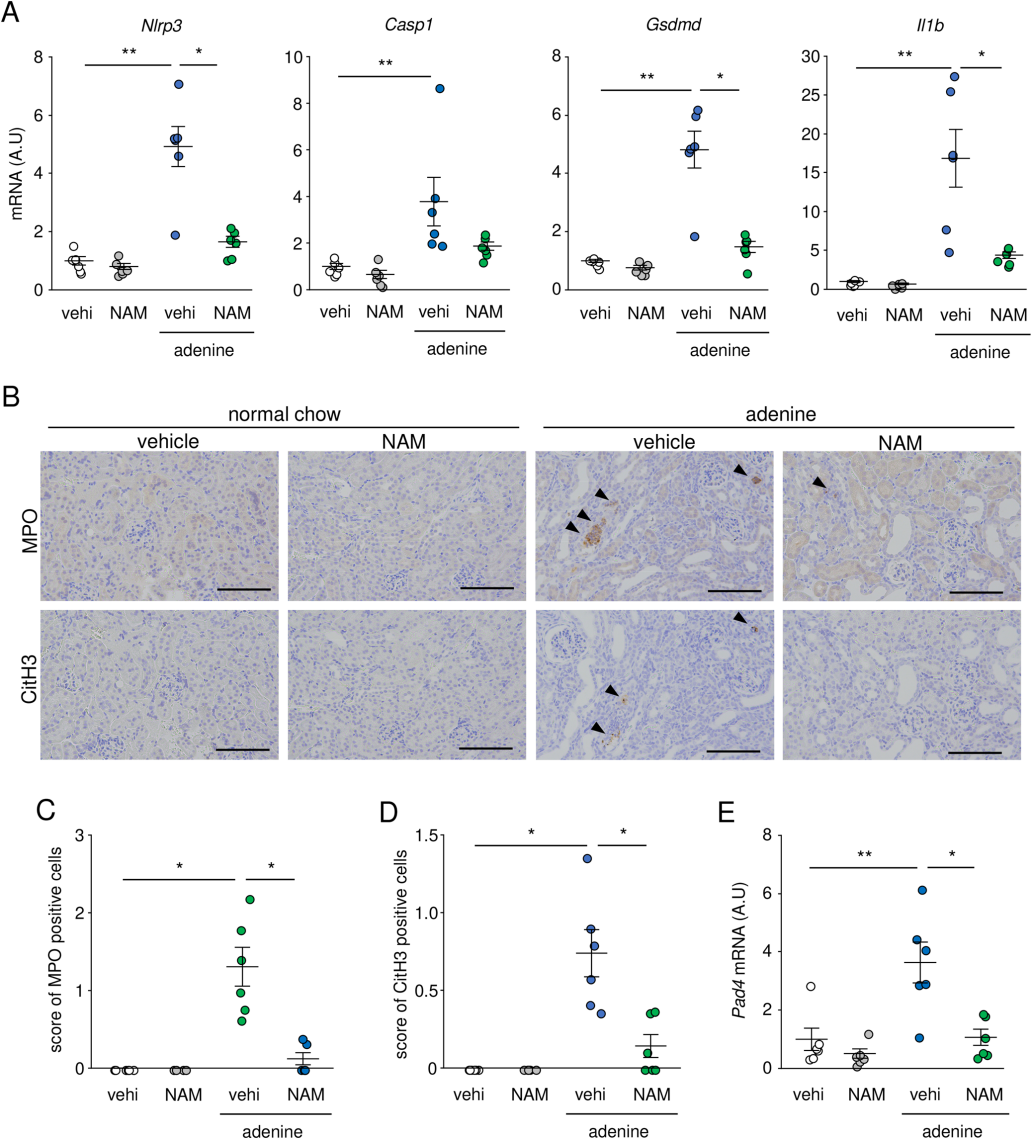
NAM reduced inflammasome and NET‐related markers in adenine‐induced nephropathy. (A) Expression levels of *Nlrp3*, *Casp1*, *Gsdmd*, and *Il1b* mRNA in the kidneys. (B) Immunohistochemistry for myeloperoxidase (MPO) and citrullinated histone H3 (CitH3). (C, D) Their infiltration scores in the kidneys. Scale bar = 100 μm. (E) *Pad4* mRNA expression in the kidneys. A.U., arbitrary units; NAM, nicotinamide; vehi, vehicle. Data are presented as mean ± SEM. *n* = 5–6. *p < 0.05, **p < 0.01.

The innate immune system, including inflammasomes and NET formation, activated the coagulation and complement cascades [29]. KEGG pathway analysis showed that the “NET formation” pathway was suppressed by NAM. Therefore, we characterized the effects of NAM on these innate immune‐related markers in the kidneys. The expression of genes related to inflammasomes and pyroptosis, including *Nlrp3*, *Casp1*, *Gsdmd*, and *Il1b* was significantly elevated in adenine‐induced nephropathy, and NAM significantly reduced the expression of *Nlrp3*, *Gsdmd*, and *Il1b* in the kidneys (Figure 5A). The infiltration of MPO‐positive cells, a neutrophil‐derived protein, was significantly reduced by NAM in adenine‐induced nephropathy (Figure 5B,C). Histone citrullination was required for NET formation and mediated by peptidyl arginine deiminase 4 (PAD4) [30]. The increased infiltration of CitH3 positive cells and the expression of *Pad4* mRNA were suppressed by NAM (Figure 5B,D,E). In addition, using a mirror‐image immunostaining method for MPO and CitH3, we observed that most CitH3‐positive cells overlapped with MPO‐positive cells (Figure S2).

### 3.7 Comparison of the Therapeutic Effect of PMX53 and NAM on Adenine‐Induced Nephropathy

To gain further insights, we compared the effects of the complement C5aR inhibitor PMX53 and NAM in adenine‐induced nephropathy. After one week of adenine treatment, we measured the expression of inflammasome‐related genes. PMX53 lowered the expression of genes such as *Nlrp3* and *Gsdmd* when administered alone, while NAM alone showed even stronger effects. Compared with NAM treatment alone, the combination with PMX53 showed no additive effect (Figure S3A,B).

## 4 Discussion

In this study, we demonstrated that (1) serum coagulation and complement marker activity or their expression in the kidneys correlate with each other in our human CKD cohort and in the Nephroseq database; (2) the complement and coagulation cascades in the kidneys are upregulated in mouse models of renal fibrosis; and (3) NAM, a NAD^+^ precursor, is a novel modulator of this cascade, inflammasomes, and NET formation in adenine‐induced nephropathy.

We first characterized the relationship between the coagulation and complement cascades in serum markers derived from patients with CKD. Our findings demonstrate that high fibrinogen levels and low PT‐INR are independently associated with high complement C3 and C4 levels, even when corrected for age, BMI, eGFR, proteinuria, and serum albumin. Previous studies have shown that C3 binds with a high affinity to fibrin(ogen) and, when incorporated into thrombi, prolongs fibrinolysis in a concentration‐dependent manner, contributing to CVD risk [31]. High plasma fibrinogen levels are an established prognostic factor for CKD progression and CVD complications [32, 33], and thereby the involvement of complement factors may underlie the pathological role of fibrinogen in CKD. Prothrombin time is an indicator that reflects the extrinsic coagulation system and tissue factor activity [34]. Other study demonstrated that shortened prothrombin time in dogs is associated with an increased incidence of thrombosis and increased circulating D‐dimers [35]. This may reflect the finding that C5a, which is activated by C5‐convertase downstream of C3, has been reported to induce tissue factor [3]. Therefore, the complement cascade may be involved in the activation of the tissue factor‐dependent extrinsic coagulation system in the pathogenesis of CKD.

Analysis of public databases showed that *FGB* and *C3* expression were positively correlated with each other and increased with pro‐fibrotic genes such as *Col1a1*. In addition, the expression of genes associated with complement and coagulation activation, such as *F3*, *Fga*, *Pai1*, *C3*, and *C5ar1*, was also elevated in the two different renal fibrosis models, indicating the upregulation of local renal coagulation and complement cascades in the pathogenesis of CKD. The expression of inducible fibrinogen in tubular cells, tissue factor, and FXa in inflammatory cells has been implicated in renal fibrosis and diabetic kidney disease [36, 37, 38, 39, 40]. Similarly, increased renal PAI1 promotes a fibrotic response in a model of UUO renal injury [41]. Furthermore, the anticoagulant low molecular weight heparin or danaparoid has been reported to decrease proteinuria in human diabetic kidney disease [42, 43]. In the complement cascade, C3 and C5 expression in tubules, C3 expression in macrophages, and increased complement synthesis in pericytes have been reported in injured kidneys [15, 44, 45]. C3 deficiency also suppresses the fibrotic response in models of renal fibrosis [45]. More critically, C3 deficiency in local kidneys protects against kidney injury, suggesting the pathological significance of complement synthesis in the kidneys [46, 47]. Furthermore, C5aR1‐mediated local inflammatory response contributes to tubulointerstitial fibrosis progression after renal ischemia reperfusion injury [48]. We also demonstrated that the C5a inhibitor reduced the expression of inflammasome‐related genes in our model. Therefore, the pathological role of increased expression of the local coagulation and complement pathway in inducing renal fibrosis has been demonstrated.

Interestingly, complement‐regulating proteins such as CD55 were suppressed in adenine‐induced nephropathy. CD55 promotes the disruption of C3 and C5 convertases, thereby inhibiting complement activation [49]. In addition, CD55 is suppressed in a renal ischemia–reperfusion model and exerts protective effects against renal injury [50, 51, 52]. CD55 expression is suppressed in NRK‐52E cells overexpressing PAX2, which may explain the mechanism by which complement‐regulating proteins are reduced in fibrotic kidneys [53].

In recent years, the association of inflammatory signaling in the innate immune system with the coagulation and complement systems has been clarified, and inflammasomes and associated cell death (i.e., pyroptosis) have been reported to upregulate tissue factor and to promote thrombotic risk [54, 55]. Moreover, the interaction of NETs and the complement and coagulation systems has become increasingly evident [56]. NAM and its downstream products are suggested to suppress innate immune responses. It has been reported that NAM and nicotinamide mononucleotide (NMN) reduce the production of cytokines such as IL1β in activated macrophages, and proteomic analysis showed that coagulation‐ and complement‐related pathways were downregulated by NMN treatment [57, 58]. In addition, supplementation with NMN to restore NAD^+^ levels, or NAD^+^ itself, has been suggested to suppress the activation of the NLRP3 inflammasome [59, 60]. On the basis of these findings, a substrate of NAD(P)H quinone oxidoreductase 1 has been demonstrated to inhibit thrombosis by hindering tumor‐derived tissue factor and NET formation through modulation of intracellular NAD^+^ levels [61]. Therefore, our study suggests that regulation of the innate immune system, including the NET and inflammasome pathways, underlies the amelioration of the prothrombotic state, excessive complement activity, and kidney injury by NAM.

One possible mechanism by which NAM ameliorates adenine‐induced nephropathy is through modulation of the complement and coagulation cascades. In contrast, comparison of the therapeutic effects of the C5aR inhibitor PMX53 and NAM revealed that NAM exerted a stronger renoprotective effect on the kidney. The beneficial actions of NAM are diverse, and the underlying mechanisms remain incompletely understood. We cannot exclude the possibility that NAM also acts through additional pathways beyond this mechanism.

Collectively, the coagulation and complement cascades were elevated in the pathogenesis of CKD and may contribute to both kidney injury and cardiovascular risks. These excess signals were suppressed by the NAD^+^ precursor NAM (Figure S4). This study identified a novel mechanism of action of NAM that may help accelerate its application in the treatment of CKD.

## Author Contributions

Y.O. conceived and designed the animal study. Y.O. and M.M. conceived and designed our CKD cohort study. S.K., Y.O., and E.S. performed experiments and analyzed data. S.K., Y.O., T.O., E.S., N.T., M.M., and T.T. interpreted the results of experiments. Y.O. drafted the manuscript and prepared figures. S.K., T.O., E.S., N.T., M.M., and T.T. edited and revised the manuscript.

## Funding

This work was supported by the Japan Society for the Promotion of Science (JSPS), 21K16156 and Gonryo Medical Foundation.

## Conflicts of Interest

The authors declare no conflicts of interest.

## Supporting information


**Data S1:** fsb271263‐sup‐0001‐Supinfo.pdf.

## Data Availability

All data and materials are available upon request. The raw sequencing data (FASTQ format) has been deposited in the DNA Data Bank of Japan (DDBJ; accession number PRJDB37909).
