# Supplementary material for: Nicotinamide Attenuates Complement and Coagulation Pathways and Resultant Renal Fibrosis
Source: FASEB J. 2025 Dec 1;39(23):e71263. doi: 10.1096/fj.202502028R (PMC12668026; doi:10.1096/fj.202502028R)
Supplement: Supplementary file 1 — Data S1: fsb271263‐sup‐0001‐Supinfo.pdf. [file FSB2-39-e71263-s001.pdf]

Supplementary information

Nicotinamide Attenuates Complement and Coagulation Pathways and Resultant Renal Fibrosis

Saori Kin[1], Yuji Oe[1], Taku Obara[2], Emiko Sato[3], Nobuyuki Takahashi[3], Mariko Miyazaki[1], Tetsuhiro Tanaka[1]

1) Department of Nephrology, Graduate School of Medicine, Tohoku University.
2) Division of Preventive Medicine and Epidemiology, Tohoku University Tohoku Medical Megabank Organization.
3) Division of Clinical Pharmacology and Therapeutics, Graduate School of Pharmaceutical Sciences, Tohoku University

#Corresponding author
Yuji Oe, M.D., Ph.D.
Department of Nephrology, Graduate School of Medicine, Tohoku University,
Sendai, 980-8574, Japan
Tel: +81-22-717-7163
E-mail: yuji.oe.b3@tohoku.ac.jp

Supplementary tables

Supplementary Table 1. Primer sequence

Gene	sequence (5'→3')	Gene	sequence (5'→3')
F3	Fw: GGAGGAGCCGCCATTTACAAA	*Cd55*	Fw: ACCTCCACTCCCAGGAAAAG
	Rv: AAACTGCTGAATTACTGGCTGT		Rv: TAGAGGAGACACCGACTAGCC
Fga	Fw: AGTCTGGACTACAGATACCGAAG	*Cd59*	Fw: GAGCCAAACAACGCAGAAACTTCC
	Rv: CGTCAATCAACCCTTTCATCCTG		Rv: GGGCATCCAGGATGACTTAGAAGC
Tfpi	Fw: TGGAGCAGAAAGGCCAGATT	*Nlrp3*	Fw: TGTGAGAAGCAGGTTCTACTCT
	Rv: TCAAAGTTGTTGCGGTTGCC		Rv: TGTAGCGACTGTTGAGGTCCA
Pai1	Fw: CAAGCTCTTCCAGACTATGGTG	*Casp1*	Fw: ACAAGGCACGGGACCTATG
	Rv: ACCTTTGGTATGCCTTTCCAC		Rv: TCCCAGTCAGTCCTGGAAATG
Plat	Fw: TGACCAGGGAATACATGGGAG	*Gsdmd*	Fw: TTCAGGCCCTACTGCCTTCT
	Rv: CTGAGTGGCATTGTACCAGGC		Rv: GTTGACACATGAATAACGGGGTT
vWF	Fw: CTTCTGTACGCCTCAGCTATG	*Il1b*	Fw: CTGTGACTCATGGGATGATGATG
	Rv: GCCGTTGTAATTCCCACACAAG		Rv: CGGAGCCTGTAGTGCAGTTG
C1qa	Fw: TTCGGCAGAACCCAATGACG	*Pad4*	Fw: TCTGCTCCTAAGGGCTACACA
	Rv: TGGTATGGACTCTCCTGGTTG		Rv: GTCCAGAGGCCATTTGGAGG
C3	Fw: CCAGCTCCCCATTAGCTCTG	*Hprt*	Fw: TCAGTCAACGGGGGACATAAA
	Rv: GCACTTGCCTCTTTAGGAAGTC		Rv: GGGGCTGTACTGCTTAACCAG
C5ar1	Fw: ACCGCCTGTATAGTCCTGC		
	Rv: GGTCGGCACTAATGGTAGCC		

Abbreviations: Fw, forward; Rv, reverse

References:

1) Sachetto, A. T. A., Jensen, J. R. & Santoro, M. L. Liver gene regulation of hemostasis-related factors is altered by experimental snake envenomation in mice. *PLoS Negl Trop Dis* **14**, e0008379 (2020). https://doi.org/10.1371/journal.pntd.0008379

2) Estrada, C. C. *et al.* Endothelial-specific loss of Krüppel-Like Factor 4 triggers complement-mediated endothelial injury. *Kidney Int* **102**, 58-77 (2022). https://doi.org/10.1016/j.kint.2022.03.025

3) Primer Bank (http://pga.mgh.harvard.edu/primerbank/)

Supplementary Table 2. Basal characteristics of our CKD cohort

	Total (n = 74)
Age (years)	53 (41-67)
Sex (Female)	29 (39.2%)
Body mass index (kg/m^2)	25.4 ± 5.1
Mean blood pressure (mmHg)	97.1 (88.7-110.1)
Hypertension	45 (60.8%)
Diabetes	14 (18.9%)
Dyslipidemia	16 (21.6%)
Findings of renal biopsy	
Nephrosclerosis	19 (25.7%)
Diabetic nephropathy	10 (13.5%)
Mes PGN	25 (33.9%)
Others	20 (27.0%)
Chemistry	
Urinary protein (g/gCre)	0.87 (0.31-1.82)
Creatinine (mg/dL)	1.04 (0.70-1.44)
eGFR (mL/min/1.73 m^2)	59.4 ± 26.8
Total protein (g/dL)	6.8 ± 0.6
Serum albumin (g/dL)	3.9 ± 0.4
Coagulation makers	
Fibrinogen (mg/dL)	311.3 ± 68.0
PT-INR	0.98 (0.95-1.01)
APTT (s)	30.5 (28.4-32.6)
Complement molecules	
C3 (mg/dL)	109.9 ± 20.1
C4 (mg/dL)	27.2 ± 6.9

Abbreviations: CKD, chronic kidney disease; Mes PGN, mesangial proliferative-glomerulonephritis; eGFR, estimated glomerular filtration rate; gCre, g creatinine; PT-INR, Prothrombin time international normalized ratio; APTT, activated partial thromboplastin time. Data are presented as mean ± SD or as median (interquartile range), as appropriate.

Supplementary figures

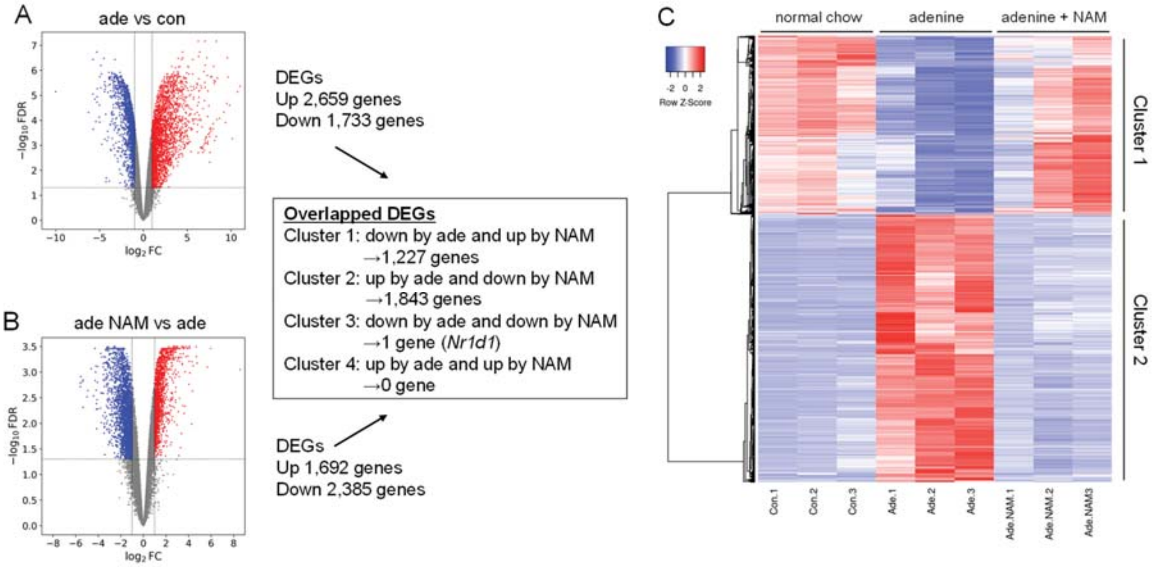

Supplementary Figure 1. RNA-seq analysis in the kidneys

Volcano plots for comparison between normal chow (con) and adenine (ade)-induced nephropathy (**A**) and between vehicle and NAM in adenine-induced nephropathy (**B**). Among the overlapping differentially expressed genes (DEGs), we detected 1227 genes that were downregulated in adenine-induced nephropathy and upregulated by NAM treatment (cluster 1). A total of 1843 genes (cluster 2) were upregulated in adenine-induced nephropathy and downregulated by NAM. **C.** A heatmap of DEGs in clusters 1 and 2 is shown. *n*=3 in each group.

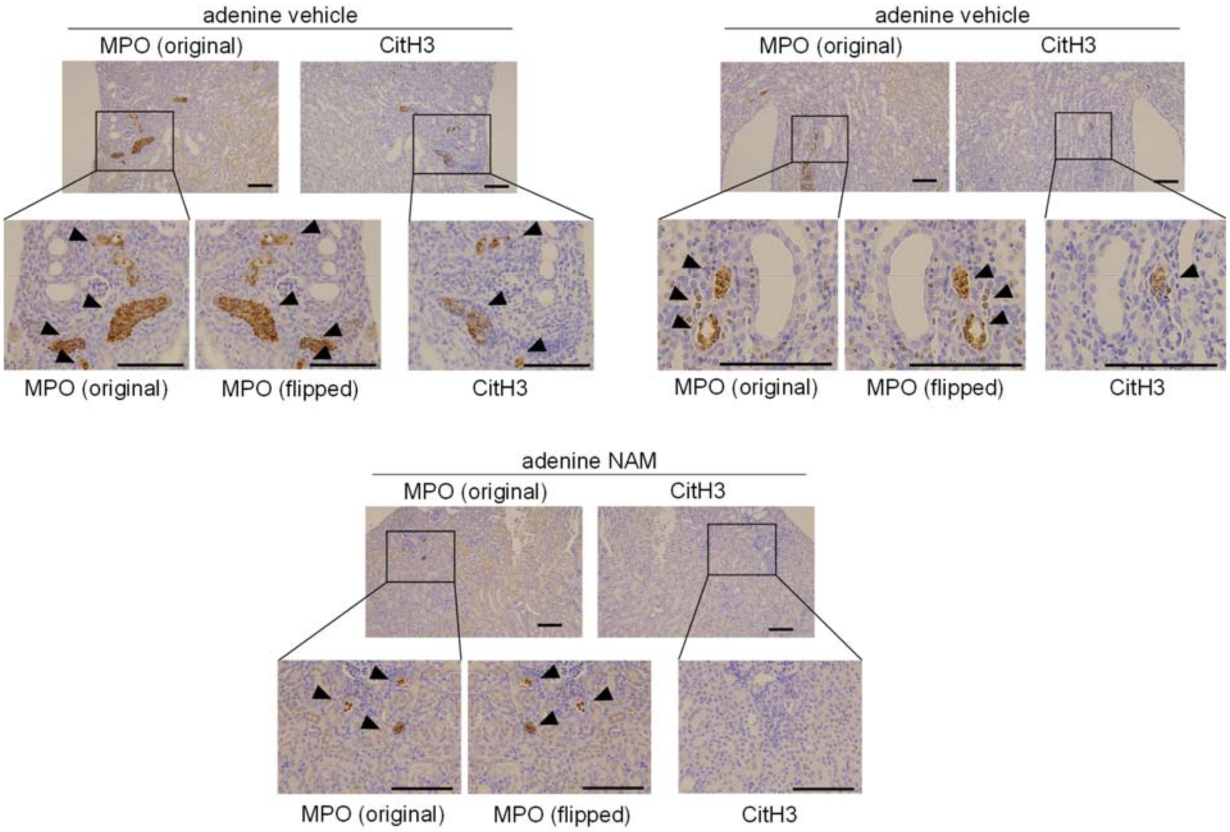

Supplementary Figure 2. Immunostaining for MPO and CitH3 using mirror sections. Representative photomicrographs showing immunohistochemical staining for myeloperoxidase (MPO) and citrullinated histone H3 (CitH3) on mirror sections. To facilitate comparison of the localization, horizontally flipped images of MPO staining are also shown. Arrowheads indicate positive cells. Scale bar = 100 μm.

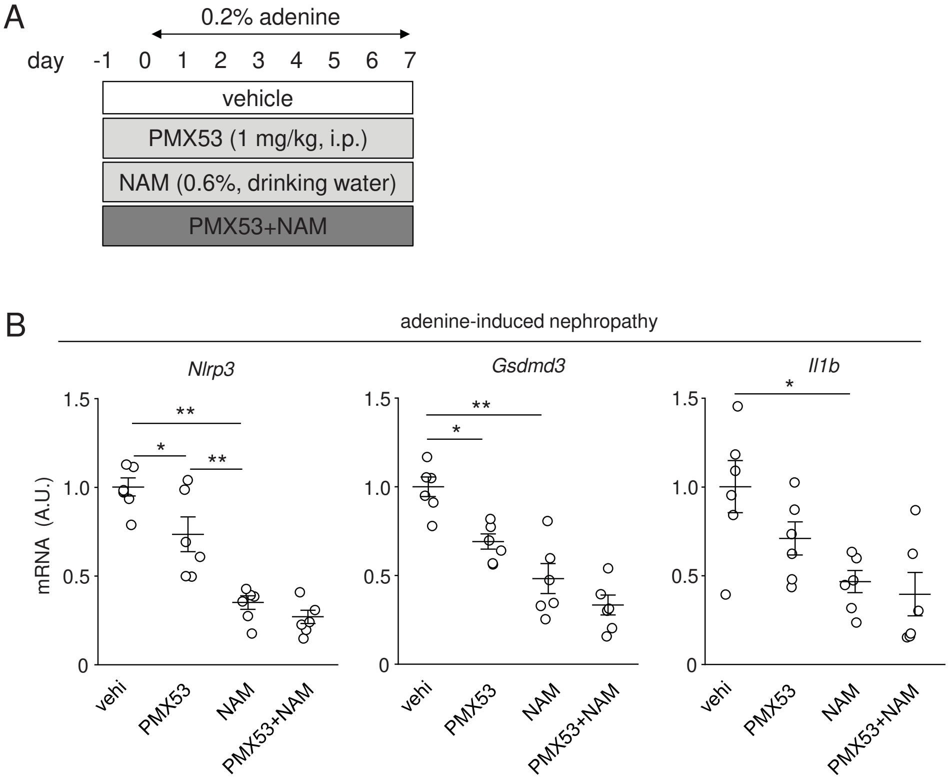

Supplementary Figure 3. Comparison of the effects of PMX53 and NAM on inflammasome-related markers.

A. An experimental protocol. **B.** Renal expression of inflammasome-related genes, including *Nlrp3*, *Gsdmd*, and *Il1b*, in adenine-induced nephropathy. i.p., intraperitoneal injection; PMX53, C5a antagonist; NAM, nicotinamide; A.U., arbitrary units. Data are presented as mean ± SEM. n = 6. *P<0.05, **P<0.01

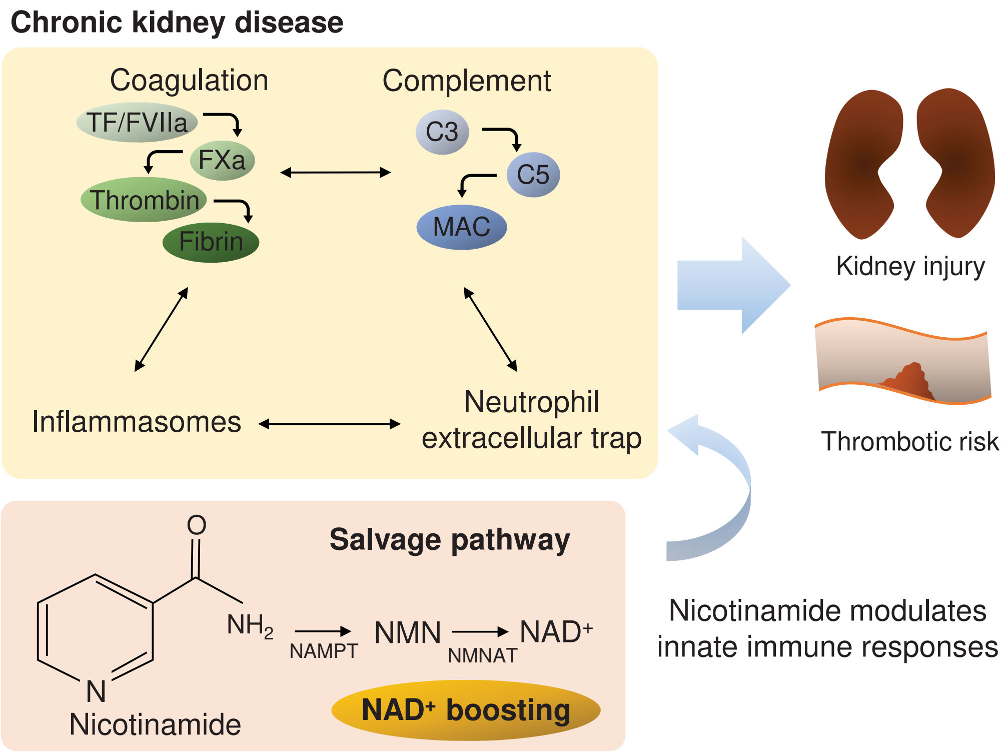

Supplementary Figure 4. Nicotinamide modulates coagulation and complement cascades in CKD Elevated coagulation and complement cascades may contribute to both kidney injury and cardiovascular risk in CKD. These excess signals and associated innate immune responses were suppressed by the NAD^+ precursor NAM. TF, tissue factor. FVIIa, factor VIIa. FXa, factor Xa. MAC, membrane attack complex. NMN, nicotinamide mononucleotide. NAMPT, nicotinamide phosphoribosyltransferase. NMNAT, nicotinamide-nucleotide adenylyltransferase.
